# Strain-Specific Antimicrobial Mechanisms of Lactic Acid Bacteria Against *Listeria monocytogenes* Scott A Under Nutrient-Rich and Carbohydrate-Limited Conditions

**DOI:** 10.3390/microorganisms14102331

**Published:** 2026-10-13

**Authors:** Spyros Didos, Konstantina Tsotsouli, Orfeas Saitis, Konstantinos Koutsoumanis, Anagnostis Argiriou

**Affiliations:** 1 Institute of Applied Biosciences, Centre for Research and Technology Hellas, 57001 Thessaloniki, Greece; sdidos@certh.gr (S.D.); ktsotsouli@certh.gr (K.T.); osaitis@certh.gr (O.S.); 2 Department of Food Science and Nutrition, University of the Aegean, 81400 Lemnos, Greece; 3 Laboratory of Food Microbiology and Hygiene, Department of Food Science and Technology, School of Agriculture, Faculty of Agriculture, Forestry and Natural Environment, Aristotle University of Thessaloniki, 54124 Thessaloniki, Greece

**Keywords:** *Listeria monocytogenes*, biopreservation, lactic acid bacteria, co-culture kinetics

## Abstract

*Listeria monocytogenes* remains a persistent food-safety challenge, sustaining interest in biopreservation with lactic acid bacteria (LAB). The mechanisms underlying LAB-mediated suppression, and their dependence on nutrient availability, remain incompletely resolved. Nine LAB isolates (four *Lactiplantibacillus plantarum*, two *Lactiplantibacillus pentosus*, three *Lactococcus lactis* subsp. *lactis*) were evaluated against *L. monocytogenes* Scott A using overlay agar-spot assays, co-culture kinetics at three effector-to-target ratios (1:1, 100:1, 10,000:1), co-culture under glucose limitation, and targeted chemical and enzymatic treatment of cell-free supernatants (CFSs). Competitive exclusion was dose-dependent: at equal inocula, most isolates permitted growth comparable to the monoculture control, whereas at 10^5^ and 10^7^ CFU/mL, pathogen growth was arrested within 6–8 h. Multivariate analysis of kinetic parameters at 10^5^ CFU/mL resolved three efficacy clusters, and efficacy tracked the onset of acidification rather than LAB growth rate: strains that lowered pH within the first 6 h achieved 31–41% inhibition, while those with an 8–10 h delay achieved <20%. *Lactiplantibacillus plantarum* DSM 20205 reached the same acidification onset as the faster-growing *Lc. lactis* strains despite a lower μ_max_, indicating higher acidifying efficiency per unit biomass. Glucose removal reduced inhibition to <6% for all *Lactiplantibacillus* isolates, whereas *Lc. lactis* BL1 and BL8 retained approximately 16%. CFS assays indicated that organic acids provide the baseline activity in nutrient-rich medium, that catalase-sensitive activity appeared in co-culture but not monoculture supernatants, and that protease-sensitive activity was present in all nine isolates under carbohydrate limitation. These results support selecting bioprotective cultures on rapid acidification combined with acid-independent mechanisms rather than on acidification alone.

## 1. Introduction

*Listeria monocytogenes* remains one of the most significant foodborne pathogens of public health concern due to its persistence in food-processing environments, psychrotrophic growth capacity, and high mortality rate associated with listeriosis [1,2,3]. Conventional control strategies, including chemical preservatives, thermal processing, and sanitizers, are increasingly challenged by consumer demand for minimally processed foods and the emergence of antimicrobial-resistant strains [2,3,4,5]. As a result, there is growing interest in biological preservation strategies, particularly the use of lactic acid bacteria (LAB) and their antimicrobial metabolites as natural and effective biocontrol agents [4,5].

Lactic acid bacteria constitute a diverse group of Gram-positive bacteria widely used in fermented foods, probiotic formulations, and biopreservation applications [6,7]. Their antagonistic activity against pathogens is multifactorial, encompassing the production of organic acids, hydrogen peroxide, diacetyl, reuterin, and a wide spectrum of bacteriocins [8,9,10]. In addition to directly inhibiting pathogen growth, LAB can modulate microbial interactions through nutrient competition, pH reduction, and immune-modulatory effects in host-associated environments [11,12,13,14]. Despite extensive research on LAB antimicrobial capabilities, the mechanistic basis of their inhibitory effects, particularly the interplay between secreted metabolites, strain-specific metabolic pathways, and environmental modulators such as nutrient availability remains incompletely understood.

Understanding the antibacterial mechanisms of LAB against *L. monocytogenes* is especially relevant because *Listeria* displays remarkable adaptability, including the ability to survive acidic conditions, osmotic stress, and various antimicrobial interventions [15,16]. Thus, identifying LAB strains with strong anti-Listerial activity and elucidating the molecular mechanisms involved are essential for the development of targeted and reliable biopreservation strategies.

To address these gaps, this study evaluates the antimicrobial potential and intrinsic competitive fitness of nine LAB strains belonging to *Lactiplantibacillus plantarum*, *Lactiplantibacillus pentosus*, and *Lactococcus lactis* subsp. *lactis*. A multi-level, environmentally dynamic approach was employed to investigate the mechanistic basis of LAB–*Listeria* antagonism. Real-time co-culture kinetics across varying effector-to-target ratios and nutrient availabilities (glucose-rich vs. substrate-depleted) were utilized to characterize population dynamics, competitive exclusion thresholds, and strain-specific metabolic efficiencies. Furthermore, targeted chemical and enzymatic treatments of cell-free supernatants (CFSs) were performed to decouple and systematically evaluate these chemical defenses, isolating the relative contributions of organic acids, oxidative stress, and proteinaceous compounds. Together, these complementary approaches offer a comprehensive evaluation of how distinct LAB strains exert targeted, environmentally dependent antibacterial effects against *L. monocytogenes*. By integrating predictive kinetic clustering with biochemical mechanistic unmasking, this study aims to advance the fundamental understanding of LAB-derived biocontrol. Ultimately, these insights will support the selection of metabolically resilient, high-efficacy starter cultures for the development of tailored, strain-specific biopreservation strategies in food systems.

## 2. Materials and Methods

### 2.1. Bacterial Strains and Inoculum Preparation

A total of nine lactic acid bacterial (LAB) strains were used in this study, including four *Lactiplantibacillus plantarum* strains, two *Lactiplantibacillus pentosus* strains, and three *Lactococcus lactis* spp. *lactis* strains (Table 1). These specific strains were selected following a preliminary screening of 16 artisanal food isolates because they exhibited the most robust in vitro antimicrobial activity against *L. monocytogenes*. Furthermore, this selection encompasses diverse ecological niches and includes the well-characterized reference strain *Lpb. plantarum* DSM 20205 to serve as a standardized benchmark for bioprotective efficacy. In addition, the pathogenic strain *Listeria monocytogenes* Scott A (Lis-2) was used as the target organism. All strains were obtained from the microbial culture collection of the Laboratory of Microbiology and Food Hygiene, School of Agriculture, Aristotle University of Thessaloniki. Stock cultures were preserved at −80 °C in Microbank™ cryogenic vials (Pro-Lab Diagnostics, Richmond Hill, ON, Canada).

To prepare working cultures, cells of *L. monocytogenes* and LAB were aseptically transferred from cryogenic beads into test tubes containing 10 mL of Tryptone Soy Broth (TSB; Lab M Limited, Lancashire, UK) and de Man, Rogosa and Sharpe (MRS) broth, respectively, and incubated at 30 °C for 24 h. The purity of each culture was verified by streaking onto Tryptone Soy Agar (TSA; Lab M Limited) for *L. monocytogenes* and MRS agar for LAB, and confirmed based on colony morphology. A single representative colony was then aseptically transferred with a sterile loop onto TSA slopes for *L. monocytogenes* and MRS agar slopes for LAB. Slopes were incubated at 30 °C for 48 h to establish the working cultures, which were subsequently stored at 4 °C and renewed every two months.

For inoculum preparation, a loopful of cells from the TSA slope culture of Lis-2 was transferred into 10 mL of TSB and incubated at 30 °C for 18 h. Similarly, LAB strains were transferred from MRS agar slopes into 10 mL of MRS broth and incubated under the same conditions. These overnight cultures were used as inoculum for the subsequent experiments.

### 2.2. Overlay Agar-Spot Assay

The antimicrobial activity of lactic acid bacteria (LAB) against *L. monocytogenes* was determined using the overlay agar-spot method, adapted from Hossain et al. [17]. Lactic acid bacteria strains were first activated and prepared as described in the previous section. Briefly, 1.5 μL of each LAB suspension (10^9^ CFU/mL) was spotted onto the surface of MRS agar plates. Plates were incubated at 30 °C for 24 h to allow visible colony development at the inoculation sites.

For preparation of the overlay, an overnight culture of *L. monocytogenes* (10^9^ CFU/mL) was diluted into Tryptone Soy Broth (TSB) soft agar (7.5 g/L agar) to yield a final concentration of 10^7^ CFU/mL. Ten milliliters of this inoculated soft agar was carefully poured over the surface of the LAB-containing MRS agar plates. The overlays were allowed to solidify at room temperature before incubation at 30 °C for 48 h.

Following incubation, inhibition of *L. monocytogenes* growth was evaluated by the appearance of clear zones surrounding the LAB colonies (spots). The diameters of the inhibition zones, including the LAB colony itself, were measured in millimeters using a ruler. Each zone was measured at least twice in perpendicular directions, and the mean value was recorded to ensure accuracy.

### 2.3. Growth Kinetics of Listeria monocytogenes in Co-Culture with LAB at Different Inoculum Levels

Bacterial growth kinetics of *L. monocytogenes* and LAB strains were assessed in both monoculture and co-culture to evaluate the effect of the initial LAB inoculum size on pathogen growth. All bacterial strains were first activated and prepared as described before. Well-grown colonies were then transferred into the corresponding broth medium and incubated at 30 °C for 18 h to obtain cultures of approximately 10^9^ CFU/mL. Each culture was harvested by centrifugation (4000× *g*, 20 min, 4 °C) and washed twice in sterile saline. The cells were then concentrated and resuspended in a minimal volume (1 mL) of sterile saline. This strict washing and concentration step was performed to completely remove residual nutrients and fermentable carbohydrates (e.g., glucose) from the activation media, thereby preventing nutrient carry-over that would otherwise compromise the subsequent glucose-limitation assays.

For co-culture assays, *L. monocytogenes* Lis-2 was inoculated at an initial concentration of 10^3^ CFU/mL. LAB strains were co-inoculated at three different initial levels, 10^3^, 10^5^, and 10^7^ CFU/mL, corresponding to low, intermediate, and high competitive pressure, respectively. Monocultures of each LAB strain and *L. monocytogenes* Lis-2 were also prepared as controls. All cultures were carried out in 100 mL BHI:MRS broth (1:1, *v*/*v*) and incubated at 30 °C for 24 h under aerobic conditions.

Samples of 1 mL were collected every 2 h up to 12 h, and again at 24 h. At each sampling point, the pH of the culture was measured. Serial decimal dilutions were prepared in phosphate-buffered saline (PBS), and viable counts were determined using the pour-plate method on selective agar media: MRS agar for LAB, and PALCAM agar (Merck KGaA, Darmstadt, Germany) for *L. monocytogenes*. Plates were incubated at 30 °C for 48 h. Colony counts were expressed as CFU/mL, and growth curves were constructed based on mean values from replicate experiments. All experiments were performed in triplicate on independent days.

### 2.4. Effect of Glucose Availability on the Antagonistic Activity of LAB Against Listeria monocytogenes

To investigate the potential effect of glucose on the antagonistic activity of LAB against *L. monocytogenes*, co-culture experiments were repeated in a glucose-free medium (Tryptone: 17 g/L, Soya peptone: 3 g/L, NaCl: 5 g/L, dipotassium phosphate: 2.5 g/L; TSB w/ο glucose). Only the intermediate initial LAB inoculum level (10^5^ CFU/mL) was selected for these glucose-free assays and subsequent multivariate clustering. This specific concentration was chosen for in-depth characterization because preliminary kinetic data showed that lower concentrations (10^3^ CFU/mL) exhibited insufficient baseline activity, while higher concentrations (10^7^ CFU/mL) caused a saturation effect that made all strains appear equally effective. Therefore, the 10^5^ CFU/mL level optimally highlighted the performance differences and significant variability among the groups, allowing us to identify the most potent candidates. Experimental procedures—including culture preparation, inoculation, sampling schedule, pH measurement, serial dilution, and plating on selective media—were carried out exactly as described for the standard BHI:MRS co-cultures. All experiments were performed in triplicate on independent days.

### 2.5. Characterization of Inhibitory Substances in Cell-Free Supernatants

To investigate the chemical nature of the inhibitory metabolites secreted by each LAB strain, filtered cell-free supernatants (CFSs) obtained from both 24-h monocultures and competitive co-cultures with *L. monocytogenes* were subjected to targeted neutralization and enzymatic treatments. Monocultures of each LAB isolate and co-cultures containing the pathogen were grown in nutrient-rich BHI:MRS (1:1, *v*/*v*) and nutrient-depleted TSB without glucose. All cultures were incubated at 30 °C for 24 h. Following incubation, the cultures were centrifuged at 4000× *g* for 20 min at 4 °C, and the resulting supernatants were collected and filtered through 0.22 μm pore-size membranes to obtain cell-free preparations. Aliquots of these unmodified, untreated supernatants were retained for direct use in antibacterial assays to establish baseline inhibitory capacity. To delineate the specific mechanisms driving pathogen suppression, the CFS preparations were subjected to differential treatments based on the growth medium. For nutrient-rich preparations (BHI:MRS), to account for inhibition driven by organic acid production, aliquots of the highly acidic BHI:MRS supernatants were neutralized to pH 6.5 using sterile 1 M NaOH. To quantify the specific contributions of oxidative stress and proteinaceous compounds, these neutralized samples were further divided and subjected to separate enzymatic treatments. To eliminate the inhibitory effects of hydrogen peroxide (H_2_O_2_), aliquots were treated with catalase (4000 Units/mg) at a final concentration of 1 mg/mL and incubated at 37 °C for 1 h [18]. To assess bacteriocin-mediated activity, separate aliquots were treated with proteinase-K (30 Units/mg) at 1 mg/mL under identical incubation conditions [19]. Following incubation, enzymes were heat-inactivated at 95 °C for 10 min to prevent residual enzyme activity from confounding pathogen growth assays. Conversely, for nutrient-depleted preparations (TSB w/o glucose), because supernatants derived from carbohydrate-restricted TSB cultures exhibited minimal metabolic acidification, pH neutralization was omitted. These non-neutralized samples were directly subjected to the parallel catalase and proteinase-K enzymatic treatments described above to isolate baseline oxidative and proteinaceous mechanisms. The antibacterial activity of all untreated, neutralized, and enzymatically treated CFS preparations was evaluated against *L. monocytogenes* using automated 96-well microplate assays. Each assay well contained a mixture of 200 μL of the respective CFS preparation and an equal volume of the corresponding fresh growth medium inoculated with the pathogen. Controls included *L. monocytogenes* cultivated under identical conditions without the addition of CFSs, as well as non-inoculated CFS-medium mixtures to serve as optical density blanks. Pathogen proliferation was monitored dynamically by measuring the optical density at 600 nm (OD_600_) over a 24-h incubation period at 30 °C. Antimicrobial efficacy was ultimately expressed as the net change in pathogen optical density (ΔOD_600_) at 24 h relative to the untreated controls. This systematic workflow enabled the evaluation and direct statistical comparison of the individual contributions of organic acids, catalase-sensitive metabolites, and putative bacteriocins to the overarching bioprotective profiles of the LAB isolates.

### 2.6. Data Analysis and Statistics

All experiments were performed in three independent biological replicates, each utilizing technical triplicates. Growth data (log CFU/mL) were fitted with the Baranyi and Roberts primary model using the DMFit Excel add-in to estimate the maximum specific growth rate (μ_max_, h^−1^) and the maximum population density (N_max_). Goodness of fit was assessed by the coefficient of determination (R^2^).

Strain inhibition (%) was defined as the percentage reduction in the final logarithmic increase in *L. monocytogenes* in co-culture relative to the monoculture control at 24 h, calculated as: I(%) = 100 × (ΔlogN_control_ − ΔlogN_co-culture_)/ΔlogN_control_.

The onset of acidification (pH_t_lag, h) was operationally defined as the first sampling time at which the culture pH had decreased by more than 0.2 units relative to the initial value. It is acknowledged that the 2-h sampling interval establishes the temporal resolution limit of this specific parameter.

Differences among strains regarding inhibition-zone diameter and ΔOD_600_ across CFS treatments were assessed by one-way ANOVA followed by Tukey’s HSD post-hoc test at *p* < 0.05, after verifying normality (Shapiro–Wilk test) and homoscedasticity (Levene’s test). Within each strain, CFS treatments were compared as an independent treatment group. Prior to calculation of the final ΔOD_600_, raw optical density values were blank-corrected against a non-inoculated CFS-medium control. Finally, kinetic parameters obtained at the 10^5^ CFU/mL inoculum level (LAB μmax, pH_t_lag, inhibition %, and pathogen μmax) were autoscaled and subjected to principal component analysis (PCA) and hierarchical clustering analysis (HCA) utilizing Euclidean distance and Ward’s linkage. All statistical analyses were performed using R version 4.2.1/IBM SPSS Statistics version 28.

## 3. Results

### 3.1. Overlay Agar-Spot Assay

All tested LAB strains exhibited antagonistic activity against *L. monocytogenes*, indicated by the formation of clear inhibition zones around the agar spots (Table 2). The degree of inhibition varied significantly among the species tested. *Lpb. plantarum* strains demonstrated the strongest antimicrobial potential, with inhibition zones ranging from 21.0 to 25.0 mm. Specifically, *Lpb. plantarum* DSM 20205 displayed the highest inhibitory activity (25 ± 1.4 mm), followed closely by strains BS14, 2640, and BS11. In contrast, *Lc. lactis* subsp. *lactis* strains (BL1, BL2, and BL8) showed the lowest inhibitory activity, with zones ranging between 13.5 and 14.0 mm. *Lpb. pentosus* strains occupied an intermediate position, exhibiting inhibition zones of approximately 16.7 to 17.3 mm. Statistical analysis confirmed that the antagonistic activity exhibited by the *Lpb. plantarum* group was significantly greater (*p* < 0.05) than that of both the *Lpb. pentosus* and *Lc. lactis* groups.

### 3.2. Growth Kinetics of Listeria monocytogenes in Co-Culture with Lactic Acid Bacteria at Different Inoculum Levels

To evaluate the competitive exclusion potential of the LAB strains under different population dynamics, the growth kinetics of *L. monocytogenes* (initial level fixed at 10^3^ CFU/mL) were monitored in co-culture with LAB strains inoculated at three different initial concentrations: 10^3^, 10^5^, and 10^7^ CFU/mL. These specific concentrations were selected to investigate inhibition efficiency at different effector-to-target ratios, ranging from an equal competition scenario (1:1 ratio, 10^3^ CFU/mL) to varying degrees of bioprotective dominance (100:1 and 10,000:1 ratios), which simulate conditions of early contamination versus the application of a high-density protective culture.

**Figure 1:**
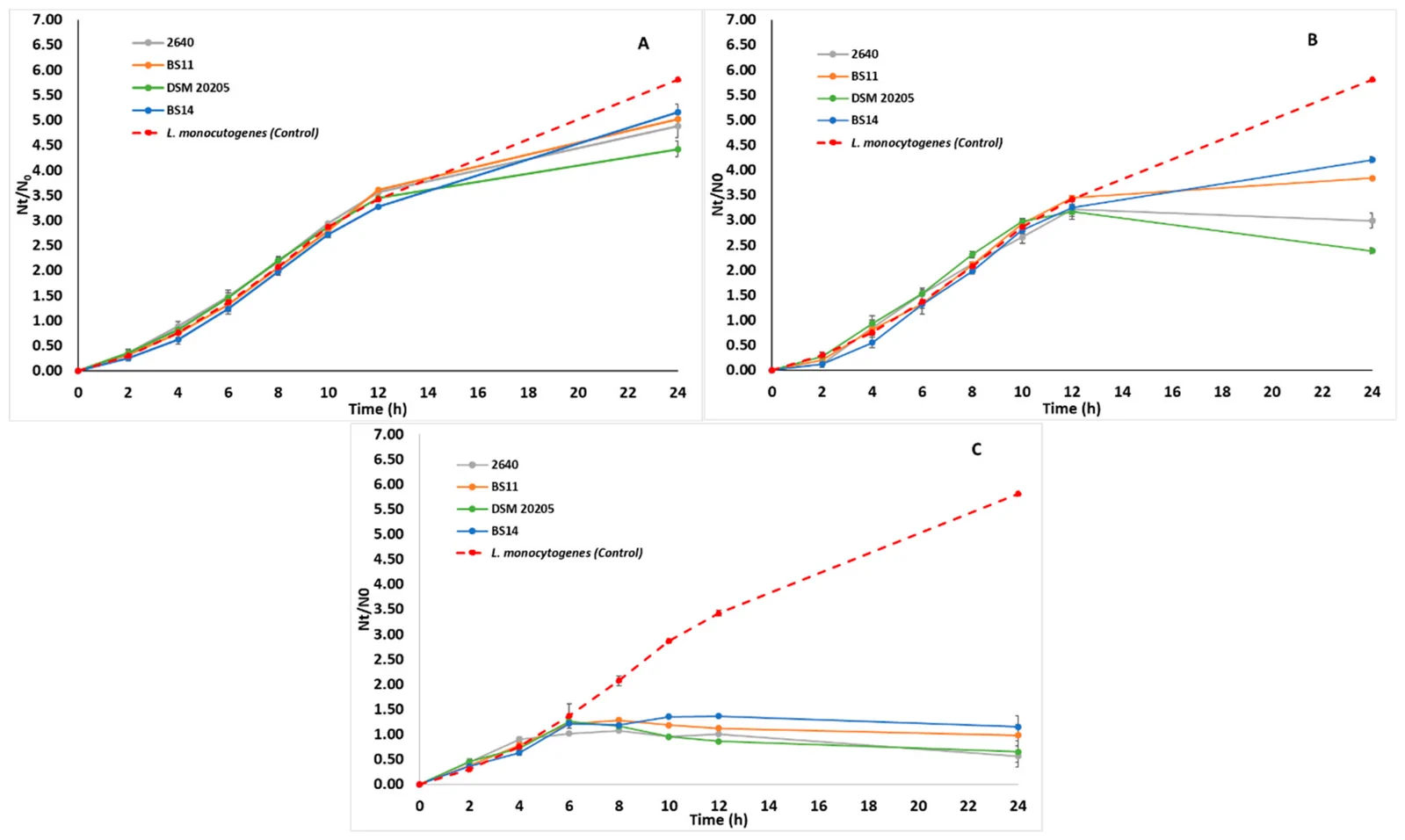
Growth kinetics of *Listeria monocytogenes* Scott A (initial level: 10^3^ CFU/mL) in co-culture with *Lactiplantibacillus plantarum* 2640, BS11, DSM 20205, and BS14 at three different initial concentrations: (**A**) 10^3^ CFU/mL, (**B**) 10^5^ CFU/mL, and (**C**) 10^7^ CFU/mL. The growth of *L. monocytogenes* monoculture was used as the control (red dashed line). Bacterial populations were expressed as the logarithmic increase in viable counts relative to the initial level (N_t_/N_0_) over 24 h at 30 °C in BHI:MRS (1:1) broth under aerobic conditions.

The inhibitory effect of *Lpb. plantarum* strains was strongly dose-dependent. In the 1:1 co-culture (10^3^ CFU/mL, Figure 1A), the growth curves of *L. monocytogenes* closely followed the control (monoculture of pathogen), reaching a final logarithmic increase (N_t_/N_0_) of approximately 4.5–5.0 after 24 h, showing minimal inhibition. However, increasing the initial LAB concentration to 10^5^ CFU/mL (Figure 1B) resulted in a noticeable deviation from the control after 10 h of incubation. By 24 h, the pathogen populations in co-culture were approximately 2 log cycles lower than the monoculture control. Notably, strain-dependent variability was most evident at this concentration; *Lpb. plantarum* DSM 20205 demonstrated the highest efficacy, maintaining the lowest pathogen numbers, whereas strain BS14 exhibited the weakest inhibition. At the highest inoculum level (10^7^ CFU/mL, Figure 1C), a bacteriostatic effect was observed; *L. monocytogenes* growth was arrested after 6–8 h, and the population increase remained below 1.5 log cycles throughout the incubation period, with *Lpb. plantarum* spp. *plantarum* DSM 20205 and *Lpb. plantarum* 2640 showing marginally stronger suppression than *Lpb. plantarum* BS11 and BS14.

**Figure 2:**
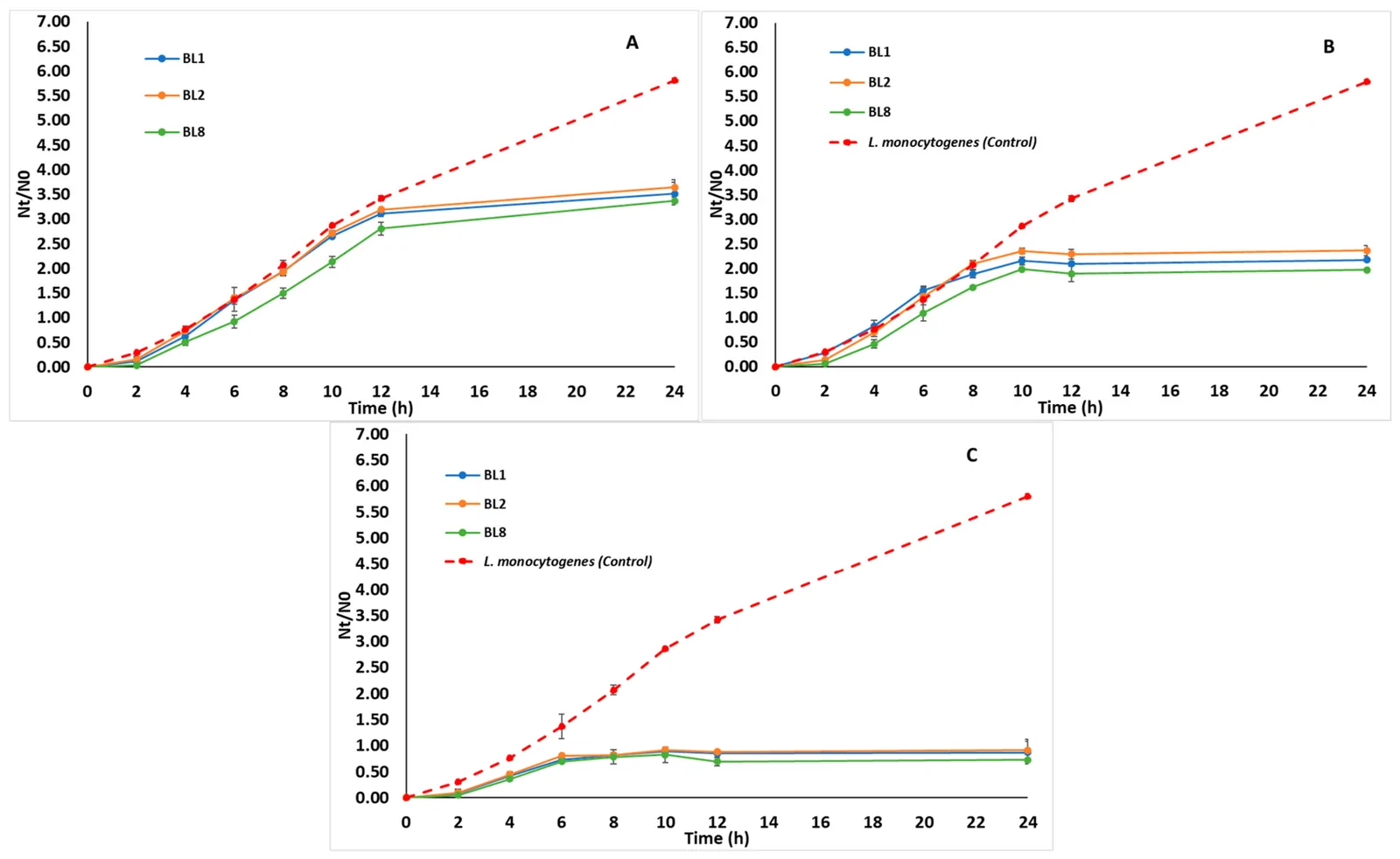
Growth kinetics of *Listeria monocytogenes* Scott A (initial level: 10^3^ CFU/mL) in co-culture with *Lactococcus lactis* subsp. *lactis* BL1, BL2 and BL8 at three different initial concentrations: (**A**) 10^3^ CFU/mL, (**B**) 10^5^ CFU/mL, and (**C**) 10^7^ CFU/mL. The growth of *Listeria monocytogenes* monoculture was used as the control (red dashed line). Bacterial populations were expressed as the logarithmic increase in viable counts relative to the initial level (N_t_/N_0_) over 24 h at 30 °C in BHI:MRS (1:1) broth under aerobic conditions.

*Lactococcus lactis* subsp. *lactis* strains exhibited the most potent inhibitory activity in liquid co-culture, particularly at lower concentrations (Figure 2). Unlike *Lpb. plantarum*, *Lc. lactis* exerted significant inhibition even at the 1:1 ratio (10^3^ CFU/mL, Figure 2A), where pathogen growth was restricted to an increase of roughly 3.5 log cycles compared to nearly 6.0 in the control. At higher inoculation levels (10^5^ and 10^7^ CFU/mL, Figure 2B,C), inhibition was immediate and profound. Strain-specific variation was particularly notable at the 10^5^ CFU/mL level; while strains BL1 and BL2 plateaued at a logarithmic increase (N_t_/N_0_) of approximately 2.2, strain BL8 demonstrated superior efficacy, restricting the increase to 1.9, a roughly 14% improvement in suppression compared to the other isolates. The N_t_/N_0_ values plateaued early (roughly 4–6 h) and remained below 2.0 (for 10^5^ inoculum) and 1.0 (for 10^7^ inoculum), indicating near-complete suppression of Listeria proliferation.

**Figure 3:**
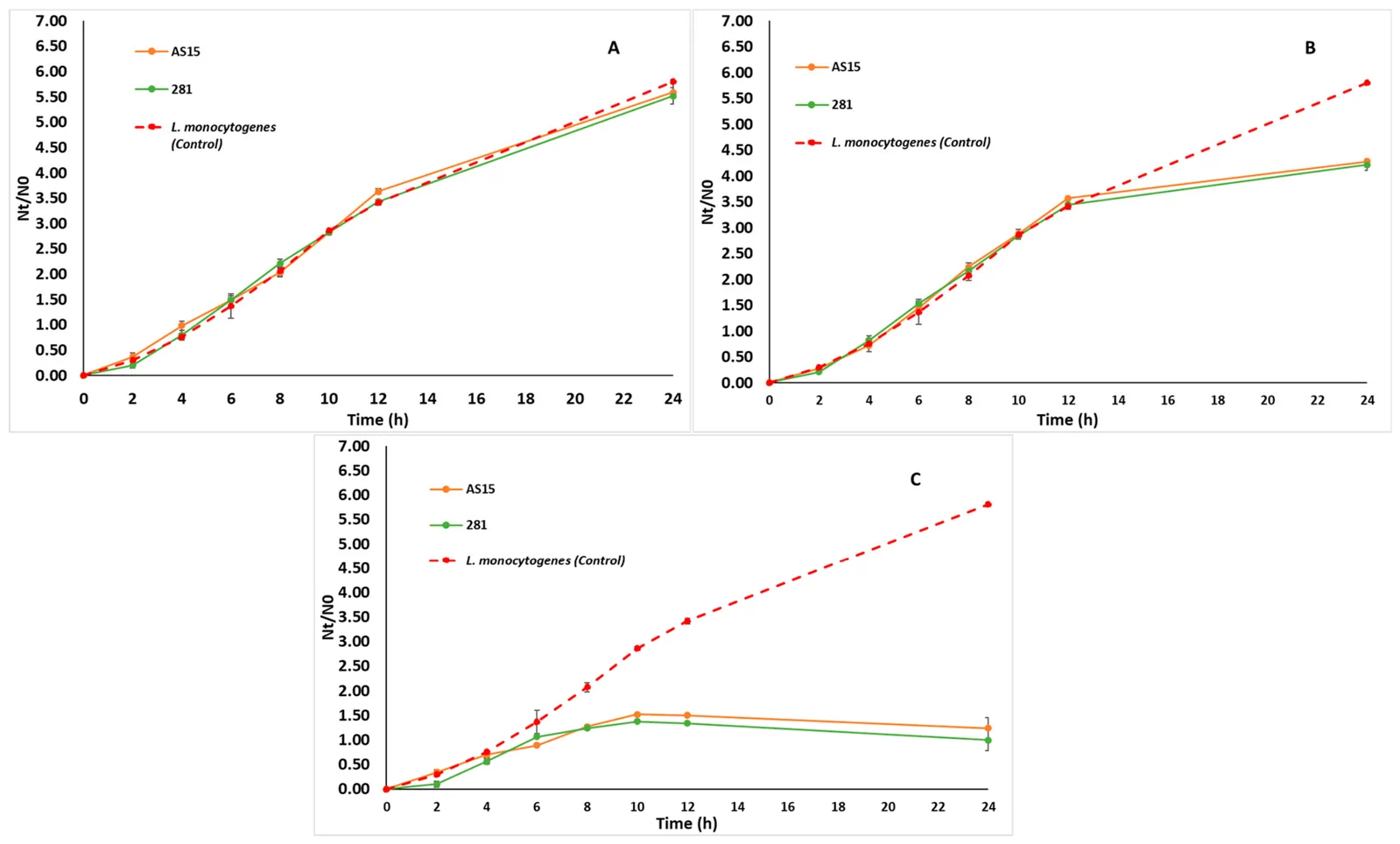
Growth kinetics of *Listeria monocytogenes* Scott A (initial level: 10^3^ CFU/mL) in co-culture with *Lactiplantibacillus pentosus* at three different initial concentrations: (**A**) 10^3^ CFU/mL, (**B**) 10^5^ CFU/mL, and (**C**) 10^7^ CFU/mL. The growth of *Listeria monocytogenes* monoculture was used as the control (red dashed line). Bacterial populations were expressed as the logarithmic increase in viable counts relative to the initial level (N_t_/N_0_) over 24 h at 30 °C in BHI:MRS (1:1) broth under aerobic conditions.

*Lactiplantibacillus pentosus* strains displayed the lowest inhibitory potential in the co-culture assay. At initial concentrations of 10^3^ and 10^5^ CFU/mL (Figure 3A,B), the pathogen growth kinetics were closely aligned with the control, suggesting that these strains require a substantial population advantage to exert an antagonistic effect. No significant differences were observed between the inhibitory profiles of strains AS15 and 281 at these lower population levels, with both exhibiting similar kinetic trends. Effective inhibition was only achieved at the highest inoculum level (10^7^ CFU/mL, Figure 3C), where the growth pattern resembled that of the *Lpb. plantarum* group, with significant suppression observed after 6–8 h of incubation. At this effective concentration, strain 281 displayed marginally higher efficacy, restricting the final pathogen increase (N_t_/N_0_) to 1.0, approximately 20% lower than the value of 1.25 observed for strain AS15.

### 3.3. Growth Kinetics of Listeria monocytogenes in Co-Culture with Lactic Acid Bacteria at Different Inoculum Levels

**Figure 4:**
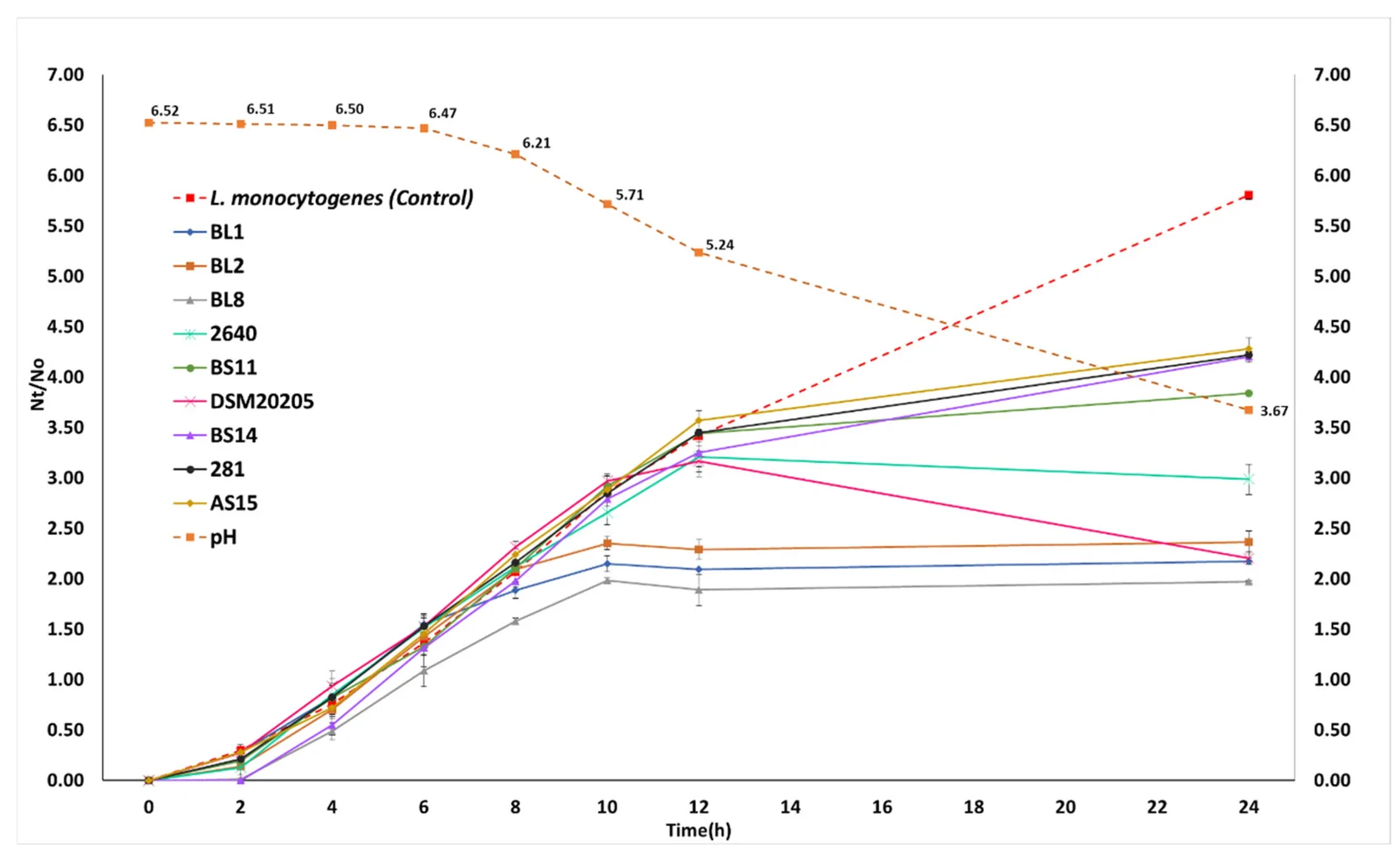
Growth kinetics of *Listeria monocytogenes* Scott A (initial level: 10^3^ CFU/mL) in co-culture with different LAB strains at initial concentration 10^5^ CFU/mL. The growth of *Listeria monocytogenes* monoculture was used as the control (red dashed line). Bacterial populations were expressed as the logarithmic increase in viable counts relative to the initial level (N_t_/N_0_) over 24 h at 30 °C in BHI:MRS (1:1) broth under aerobic conditions.

The comparative analysis of growth kinetics at the 10^5^ CFU/mL inoculum level established a clear hierarchy of antimicrobial efficacy among the studied LAB groups (Figure 4). The entire *Lc. lactis* group (BL1, BL2, and BL8) consistently exhibited the most potent and rapid suppression of *L. monocytogenes*, maintaining the lowest pathogen populations throughout the incubation period. *Lactiplantibacillus plantarum* strains displayed marked within-species variability; while *Lpb. plantarum* DSM 20205 emerged as a highly effective isolate with an inhibitory profile comparable to the *Lactococcus* group (N_t_/N_0_ ≈ 2.4), the remaining *Lpb. plantarum* strains and all *Lpb. pentosus* isolates showed significantly reduced efficacy. Collectively, these results confirm that at this intermediate concentration, pathogen control is strain-specific rather than determined solely by population size, identifying the *Lc. lactis* isolates and *Lpb. plantarum* DSM 20205 as the most promising candidates for biopreservation in liquid systems.

**Figure 5:**
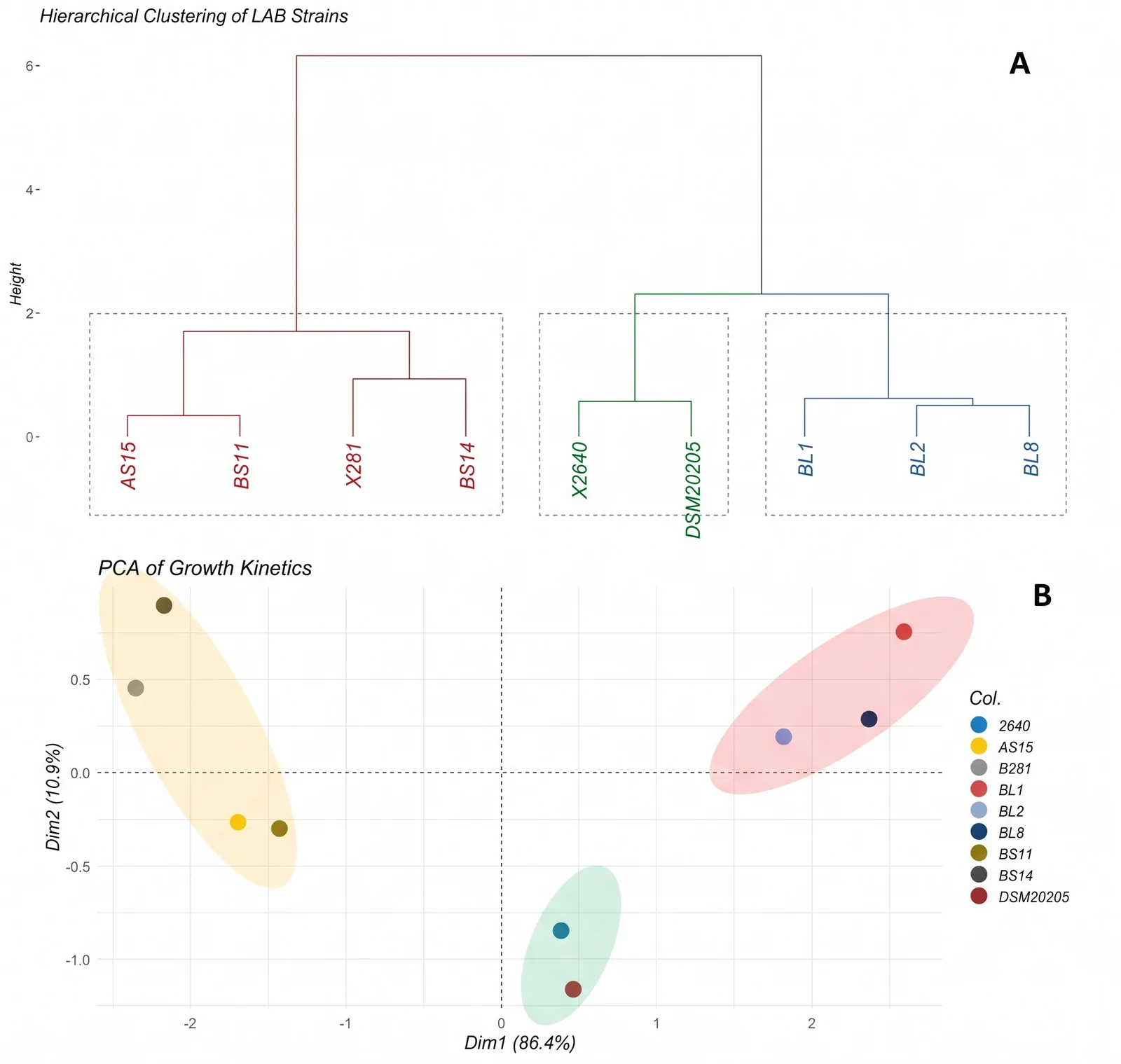
Multivariate statistical analysis of LAB growth kinetics and inhibition parameters. (**A**) Hierarchical Clustering Analysis (HCA) dendrogram classifying strains into three functional efficacy groups. (**B**) Principal Component Analysis (PCA) biplot of the kinetic and inhibition parameters.

**Figure 6:**
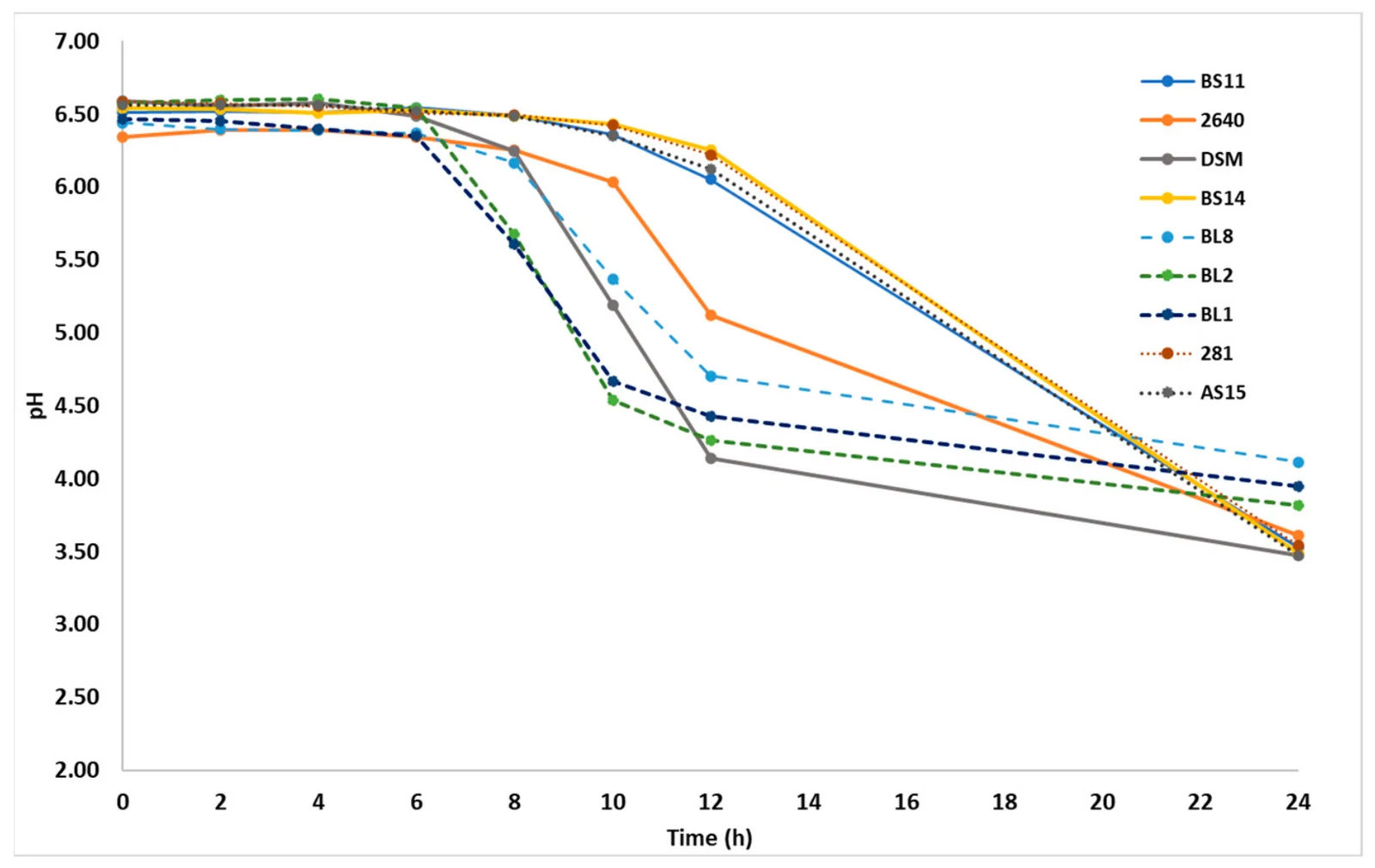
Evolution of pH during co-culture of *Lactiplantibacillus plantarum*, *Lactiplantibacillus pentosus*, and *Lactococcus lactis *strains with *Listeria monocytogenes* Scott A at an initial Lactic acid bacteria inoculum level of 10^5^ CFU/mL. Measurements were taken every 2 h up to 12 h, and at 24 h. Results represent mean values from triplicate independent experiments.

To elucidate the strain-dependent variability observed at the 10^5^ CFU/mL inoculum level, the kinetic parameters were subjected to Principal Component Analysis (PCA) and Hierarchical Clustering Analysis (HCA). The HCA dendrogram (Figure 5B) revealed the segregation of the LAB strains into three descriptive groups based on their antimicrobial performance. However, because this analysis is based on a limited panel of nine isolates, these groupings serve primarily as a comparative framework within the context of this study rather than a broad biological classification. Cluster I, designated as the high-efficacy group, comprised exclusively *Lc. lactis* subsp. *lactis* strains (BL1, BL2, BL8). This group exhibited the highest inhibition percentages (35–40%) and the fastest specific growth rates (μ_max_ ≈ 1.14–1.25 h^−1^) during co-culture (Table S1). Cluster II, categorized as the moderate-/high-efficacy group, was formed by *Lpb. plantarum* strains DSM 20205 and ACA-DC 2640. These strains clustered closely to the *Lactococcus* group in the PCA biplot (Figure 5A), distinguishing themselves from other *Lactiplantibacilli* by their superior ability to suppress pathogen growth (approx. 31–36% inhibition). Cluster III, identified as the low-efficacy group, included all *Lpb. pentosus* strains (281, AS15) and the remaining *Lpb. plantarum* isolates (BS11, BS14), and was characterized by significantly lower inhibition values (<20%). The mechanism driving this clustering was further characterized by analyzing the acidification profiles (Figure 6). A clear relationship was identified between the onset of acidification (pH_t_lag) and the suppression of *L. monocytogenes*. The most effective strains, Cluster I and Cluster II, initiated a rapid pH decline, with the onset of acidification occurring at 6 h. This early acidification occurred during the pathogen’s exponential growth phase, effectively suppressing its further growth. In contrast, the low-efficacy Cluster III strains exhibited a delayed pH drop (pH_t_lag ≈ 8–10 h). This delay allowed *L. monocytogenes* to maintain a high growth rate (μ_max_ ≈ 0.33 h^−1^) for a longer duration before inhibitory conditions were established. Notably, *Lpb. plantarum* DSM 20205 achieved comparable inhibition (35.9%) and early acidification (6 h) despite a moderate growth rate (μmax = 0.84 h^−1^). This suggests a potentially high rate of acidogenesis relative to its biomass accumulation compared to the other *Lpb. plantarum* strains; however, direct quantification of organic acids would be required to confirm this metabolic efficiency.

### 3.4. Effect of Glucose Availability on Antagonistic Activity

**Figure 7:**
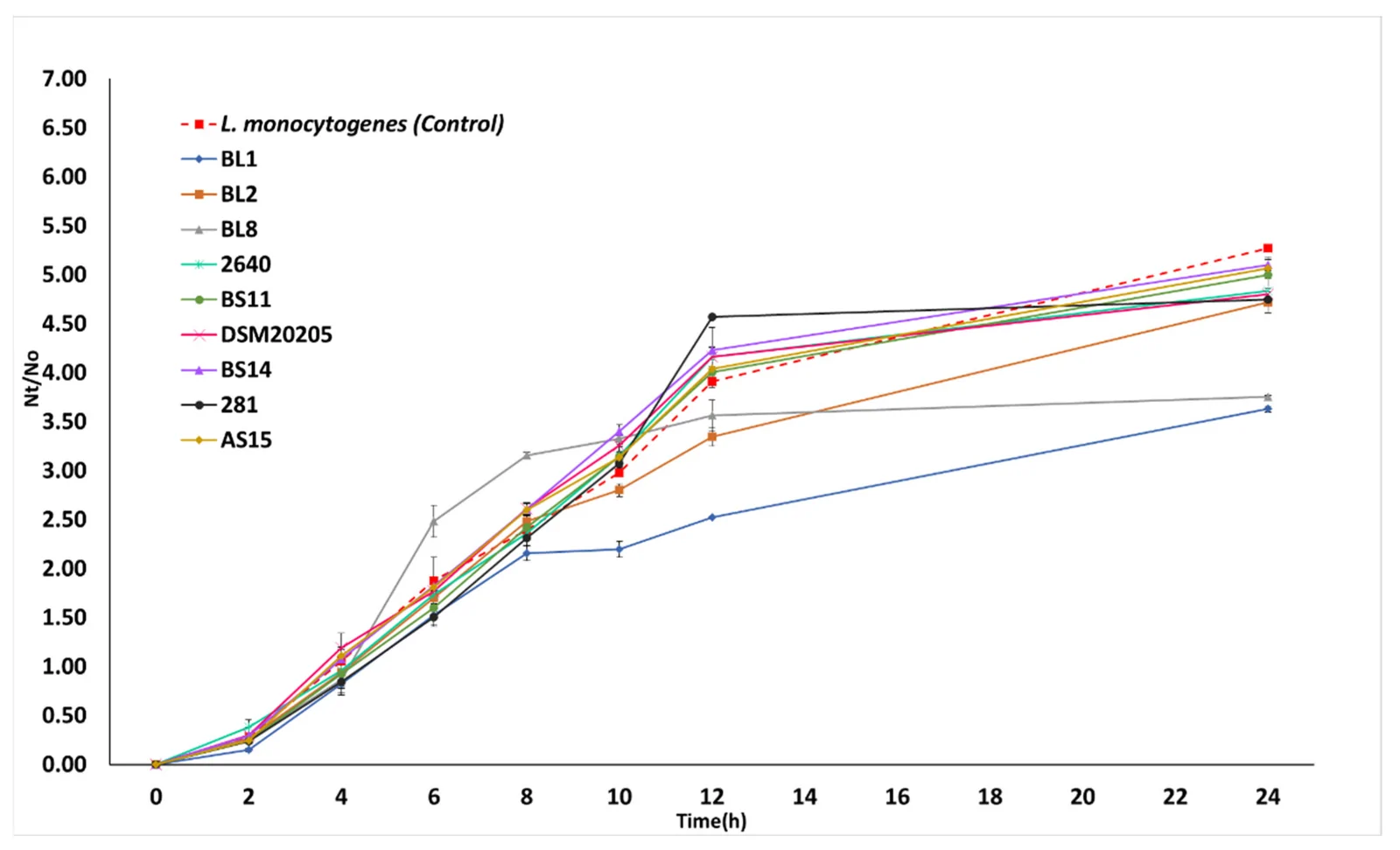
Growth kinetics of *Listeria monocytogenes* Scott A (initial level: 10^3^ CFU/mL) in co-culture with different LAB strains at initial concentration 10^5^ CFU/mL. The growth of *L. monocytogenes* monoculture was used as the control (red dashed line). Bacterial populations were expressed as the logarithmic increase in viable counts relative to the initial level (N_t_/N_0_) over 24 h at 30 °C in TSB w/o glucose broth under aerobic conditions.

To investigate the metabolic dependency of the observed antagonism, co-culture experiments were repeated in a glucose-free medium (TSB w/o glucose) using the intermediate LAB inoculum level (10^5^ CFU/mL). The absence of glucose significantly altered the competition dynamics, leading to a general reduction in the inhibitory capacity of the LAB strains compared to the glucose-rich environment. However, the extent of this reduction was highly strain-dependent, further amplifying the variability in bioprotective potential (Figure 7).

*Lactococcus lactis* strains retained the highest relative efficacy under glucose-limiting conditions. Specifically, strains BL1 and BL8 maintained a significant suppressive effect, restricting the pathogen’s specific growth rate (μ_max_) to 0.35 h^−1^ and 0.805 h^−1^, respectively, and achieving the highest inhibition percentages of approximately 16–17% (Table S2). In contrast, strain BL2 showed a marked loss of activity, with inhibition dropping to 4.26%, diverging significantly from the other *Lactococcus* strains.

*Lactiplantibacillus plantarum* strains exhibited a substantial decline in antagonistic activity. DSM 20205, which was highly effective in the glucose-rich medium, showed a drastic reduction in efficacy, with inhibition falling to just 5.09%. Similarly, strain 2640 showed limited activity (4.33%), while BS11 and BS14 exerted negligible inhibition (<3%), allowing *L. monocytogenes* to grow at rates nearly identical to the monoculture control.

The *Lpb. pentosus* group remained the least effective, with strains AS15 and 281 showing minimal inhibition (1.09% and 4.24%, respectively). The kinetic analysis (Table S2) confirmed that in the absence of glucose, most LAB strains failed to sufficiently reduce the maximum growth rate of the pathogen *L. monocytogenes*, which ranged from 0.39 to 0.745 h^−1^, indicating that the primary mechanism of inhibition, likely organic acid production, was severely compromised by substrate limitation. Collectively, these results highlight that glucose availability is a critical modulator of LAB antagonism, with *Lc. lactis* strains BL1 and BL8 demonstrating superior metabolic resilience compared to the *Lactobacillus* species.

The integration of kinetic parameters from both experimental conditions (Tables S1 and S2) provides compelling evidence regarding the mechanism of action. A direct comparison reveals that the suppression of *L. monocytogenes* is strictly metabolically driven rather than a result of simple nutrient competition. In the glucose-rich environment, the most effective strains, *Lc. lactis* (BL1, BL8) and *Lpb. plantarum* DSM 20205, achieved robust inhibition (35–40%) by severely depressing the pathogen’s specific growth rate (μ_max_ ranged from 0.19 to 0.30 h^−1^). However, under glucose depletion, the suppression of *L. monocytogenes* was severely compromised. Despite the general nutrient limitation in the glucose-free medium, the pathogen’s growth rate actually increased significantly (0.35–0.80 h^−1^) compared to the inhibitory control.

This reliance confirms that organic acid production is the dominant antagonistic mechanism. However, *Lc. lactis* subsp. *lactis* strains (BL1 and BL8) exhibited a unique metabolic resilience, retaining approximately 16% inhibitory activity even in the absence of glucose. This suggests that these strains employ a multi-factorial defense strategy, likely combining rapid acid production with secondary mechanisms such as nutrient competition or bacteriocinogenesis. Consequently, *Lc. lactis* BL1 and BL8 emerge as the superior candidates for biopreservation, offering a multi-mechanistic defense that remains partially effective even under substrate-limited conditions.

### 3.5. Mechanistic Characterization of Antimicrobial Activity in Cell-Free Supernatants

To comprehensively investigate the chemical nature of the inhibitory metabolites produced by the LAB strains, CFSs harvested from 24-h monocultures and co-cultures with *L. monocytogenes* were subjected to targeted neutralization and enzymatic treatments. Antimicrobial efficacy was quantified by measuring the total change in pathogen optical density (ΔOD_600_) after 24 h of incubation compared to the untreated pathogen control. By systematically neutralizing organic acids (pH adjustment to 6.5), destroying hydrogen peroxide (catalase treatment), and digesting proteinaceous compounds (proteinase-K treatment), the relative contributions of these distinct antimicrobial mechanisms were isolated across nutrient-rich and nutrient-depleted matrices.

#### 3.5.1. Acidification and Inducible Oxidative Stress in Nutrient-Rich Media

In the carbohydrate-rich BHI:MRS (1:1) medium (Figure S1), the primary baseline defense for all evaluated strains was robust organic acid production. The unmodified, non-treated CFS of every isolate completely arrested *L. monocytogenes* growth (ΔOD_600_ values ranging strictly from 0.01 to 0.03), demonstrating maximal baseline bioprotective capacity. Simple pH neutralization significantly reversed this inhibition across all samples, restoring pathogen growth to absolute ΔOD_600_ levels between 0.61 and 0.81, confirming that acidification is the foundational mechanism of pathogen suppression under optimal fermentation conditions However, a critical mechanistic divergence was revealed when comparing the neutralized treatments of co-cultures versus monocultures, suggesting the presence of a potentially inducible oxidative defense system. Among the “Oxidative Group”, comprising all *L. lactis* strains (BL1, BL2, BL8) and specific *Lactobacillus* isolates (*Lpb. plantarum* BS11, BS14; *Lpb. pentosus* 281), pH neutralization of the co-culture CFS only partially restored pathogen growth (ΔOD_600_ ≈ 0.61 to 0.69). Full or significantly enhanced restoration of the pathogen growth was achieved only when the neutralized supernatant was concurrently treated with catalase, driving pathogen recovery up to ΔOD_600_ levels spanning 0.82 to 0.96. For example, in *Lc. lactis* BL2 co-cultures, destruction of peroxide caused pathogen recovery to surge from 0.61 (neutralized alone) to 0.96 (neutralized + catalase). This statistically significant upward shift suggests that these specific strains may produce catalase-sensitive metabolites (likely H_2_O_2_) during direct competition with the pathogen, acting synergistically with organic acids. Crucially, these patterns imply that this oxidative response may be inducible rather than constitutive. When the same oxidative strains were cultivated in monoculture (absence of *L. monocytogenes*), the addition of catalase to the neutralized CFS provided no statistically significant growth advantage to the pathogen compared to neutralization alone, with both conditions yielding nearly identical final yields (ΔOD600 ≈ 0.78 to 0.84). The observation that these strains do not appear to accumulate effective levels of catalase-sensitive compounds when growing in isolation suggests that this mechanism might be actively triggered by the competitive co-culture environment. However, direct chemical quantification of H_2_O_2_ is required to confirm this inducible response. Conversely, *Lpb. plantarum* DSM 20205, *Lpb. plantarum* 2640, and *Lpb. pentosus* AS15 relied strictly on acidogenesis, as their co-culture CFSs showed no significant difference between the neutralized and catalase-treated samples (e.g., *Lpb. plantarum* DSM 20205 remaining static at ΔOD_600_ ≈ 0.61 for both treatments), indicating a lack of active peroxide deployment.

#### 3.5.2. Unmasking Constitutive Proteinaceous Defenses in Nutrient-Depleted Media

To evaluate the antimicrobial capabilities of the strains when primary glycolytic fermentation is restricted, CFS assays were conducted using supernatants from TSB without glucose (Figure S2). Under these carbohydrate-starved conditions, the production of organic acids and lethal oxidative metabolites was metabolically limited, allowing the untreated CFS to permit minor baseline pathogen growth (ΔOD_600_ values ranging from 0.06 to 0.12). Consequently, supplemental catalase treatment yielded no distinct recovery effect, leaving final optical densities virtually unchanged (ΔOD_600_ ≈ 0.05 to 0.11). Remarkably, targeted enzymatic digestion unmasked a highly significant secondary defense mechanism. For every evaluated LAB isolate, treatment of the starvation CFS with proteinase-K resulted in a dramatic, statistically significant restoration of *L. monocytogenes* growth. In the co-culture assays, the proteinase-K treatments consistently separated into statistically higher groupings compared to their respective non-treated baselines. For instance, robust enzymatic reversal was observed in *Lc. lactis* BL2 co-cultures, where pathogen recovery escalated from a baseline of 0.12 to 0.34 upon peptide cleavage. An even more pronounced jump occurred in *Lpb. plantarum* DSM 20205, where proteinase-K digestion released the pathogen to reach a final yield of 0.54, up from a suppressed baseline of 0.06. Furthermore, an identical statistical pattern of proteinase-K reversal was observed in the supernatants harvested from standard monocultures in TSB without glucose, driving final pathogen recovery from baseline levels of approximately 0.10 up to robust yields spanning 0.34 to 0.58 across the collection. This universal enzymatic neutralization indicates that the synthesis of protease-sensitive inhibitory compounds (bacteriocin-like inhibitory substances, BLIS) is a constitutive physiological trait across all nine isolates. Unlike the inducible oxidative defense, these protease-sensitive compounds appear to be continuously secreted as a baseline chemical shield, ensuring the strains retain potent anti-*Listeria* capability even in severe nutritional deficits where standard fermentation pathways are halted.

## 4. Discussion

### 4.1. Baseline Antagonism and Methodological Considerations

In the present study, all tested LAB strains exhibited antagonistic activity against *L. monocytogenes* during the preliminary overlay agar-spot assay. Among the isolates, *Lpb. plantarum* strains demonstrated the strongest antimicrobial potential, producing inhibition zones ranging from 21.0 to 25.0 mm. These findings align with previous studies [20,21,22] and confirm that *Lpb. plantarum* acts as a potent antagonist, likely due to the combined production of organic acids and stable bacteriocins, such as plantaricins [23].

Significant variations in efficacy were observed across the other species (*p* < 0.05). While *Lpb. pentosus* exhibited intermediate activity, *Lc. lactis* strains unexpectedly displayed the smallest inhibition zones (13.5–14.0 mm). Although *Lc. lactis* is renowned for producing nisin, a highly potent, broad-spectrum bacteriocin [24], the smaller zones observed in this study can be attributed to the physicochemical properties of the antimicrobial metabolites. Unlike small organic acid molecules that diffuse rapidly through solid media, larger or hydrophobic peptides frequently face severe diffusion limitations in agar matrices, resulting in localized zones that underestimate their true biological potency [25,26].

Crucially, the selection of the overlay agar-spot method over cell-free supernatant (CFS) well diffusion was vital for initial detection. Preliminary screening using CFS well diffusion yielded false negatives across all strains, a discrepancy consistent with the literature suggesting that well diffusion assays often fail due to insufficient antagonist concentrations in standard supernatants [27,28]. The agar-spot method proved superior because it utilizes live, metabolically active cells, allowing for the continuous synthesis, localized accumulation, and direct secretion of antimicrobial substances during co-incubation [29,30].

### 4.2. Population Dynamics and Dose-Dependent Competitive Exclusion

To overcome the spatial limitations of agar matrices, liquid co-cultivation was employed, revealing that competitive exclusion by the evaluated LAB isolates is strictly dose-dependent. The kinetic growth profiles of *L. monocytogenes* established the initial effector-to-target ratio as the primary determinant of bioprotective efficacy. In equivalent co-culture scenarios (1:1 ratio; 10^3^ CFU/mL), the majority of *Lpb. plantarum* and *Lpb. pentosus* strains permitted pathogen growth that closely mirrored the monoculture control. This aligns with broad consensus in predictive food microbiology, which dictates that at equivalent or low inoculum ratios, bioprotective cultures frequently fail to exert sufficient environmental pressure to truncate the early exponential growth phase of *Listeria* [31,32,33,34].

Significant suppression across these standard fermentative isolates was only achieved when the LAB possessed a numerical advantage of at least 100:1 (10^5^ CFU/mL) or 10,000:1 (10^7^ CFU/mL). At these elevated concentrations, pathogen kinetics were heavily truncated, exhibiting a classic bacteriostatic effect. This phenomenon is driven by the “Jameson effect,” a well-documented ecological interaction wherein the growth of a suppressed sub-population is forced into the stationary phase as soon as the dominant competitor reaches its maximum population density, N_max_ [35,36,37]. By inoculating the LAB at 10^5^ or 10^7^ CFU/mL, the strains reached their N_max_ threshold much earlier in the incubation period. Consequently, while the specific growth rate (μ_max_) of *Listeria* may remain unaffected during the earliest hours of fermentation, its final maximum population density is drastically reduced, yielding the 2-log reductions observed in our assays, a kinetic shift consistent with predictive models established by Costa et al. and Serra-Castelló et al. [33,38].

### 4.3. Strain-Specific Fitness and Metabolic Efficiency

While high inoculum levels universally induced early stationary phase onset for the pathogen, utilizing an intermediate inoculum level (10^5^ CFU/mL) was critical for unmasking the intrinsic competitive fitness of the isolates. By avoiding the masking effects of insufficient competition at low doses or artificial saturation at high doses, the multivariate kinetic analysis (PCA and HCA) demonstrated that bioprotective efficacy is fundamentally strain-specific. The segregation of strains into highly effective (>31% inhibition) and ineffective (<20% inhibition) groups aligns strongly with recent large-scale screening studies, which emphasize that elite bioprotection is a rare, strain-level trait rather than a broad species characteristic [39,40,41].

Our kinetic clustering linked high bioprotective efficacy to the rapid onset of acidification. The superior inhibitory performance of Clusters I (*Lc. lactis* strains) and II (*Lpb. plantarum* DSM 20205) was defined by their ability to induce a rapid pH drop within the first 6 h of co-culture (pH_t_lag ≈ 6 h), stressing the pathogen precisely as it entered exponential growth. Conversely, strains with a delayed pH drop (8–10 h) failed to control the pathogen. This temporal dynamic corroborates the prevailing consensus that rapid organic acid production is the most decisive antimicrobial mechanism of LAB in food systems. Stronger pathogen suppression is consistently observed when LAB rapidly lower environmental pH, a kinetic phenomenon validated across diverse food matrices [42,43].

Perhaps the most compelling mechanistic insight from this kinetic clustering is the decoupling of raw cellular growth rate from bioprotective efficacy, prominently demonstrated by *Lpb. plantarum* DSM 20205. While the highly effective *Lc. lactis* strains achieved rapid acidification via aggressive biomass accumulation (μ_max_ ≈ 1.14–1.25 h^−1^), strain DSM 20205 achieved the exact same early acidification onset and comparable inhibition (35.9%) despite a significantly slower growth rate (μ_max_ = 0.84 h^−1^). This observation suggests that DSM 20205 may possess a highly efficient acidifying metabolism. While direct analytical quantification of organic acids is necessary to confirm this, we hypothesize that, rather than utilizing energy primarily for rapid cellular replication, this strain might heavily sour the matrix with comparatively less biomass. Such a physiological advantage is increasingly recognized as a hallmark of elite bioprotective starter cultures [43,44].

### 4.4. Metabolic Dependency and Resilience Under Substrate Limitation

To test the robustness of this acid-driven inhibition, co-cultures were subjected to glucose-depleted conditions (TSB w/o glucose). The resulting collapse in LAB inhibitory capacity indicated that the suppression of *L. monocytogenes* is a chemically driven metabolic process rather than simple spatial competition. The near-total loss of bioprotective efficacy in the *Lpb. plantarum* isolates (including the previously elite *Lpb. plantarum* DSM 20205, which dropped to approx. 5% inhibition) perfectly illustrates the physiological limitations of obligate acidifiers. Without fermentable carbohydrates, these strains cannot accumulate sufficient lactic acid to execute the rapid pH decline necessary to arrest *Listeria* growth, rendering them highly vulnerable to matrix composition [45,46].

Despite the general failure of the *Lactobacillus* species under substrate limitation, the unique metabolic resilience exhibited by *Lc. lactis* subsp. *lactis* strains BL1 and BL8 represents a highly significant physiological divergence. By retaining over 16% inhibition and heavily depressing the pathogen’s specific growth rate, these strains demonstrated the capacity to seamlessly pivot to alternative, carbohydrate-independent antimicrobial strategies. The literature confirms that while acid production plummets during starvation, specific *Lc. lactis* strains possess high metabolic plasticity, selectively maintaining the synthesis of proteinaceous antimicrobial compounds (BLIS) [47,48] or shifting central carbon flux toward oxidative metabolism to accumulate inhibitory compounds like hydrogen peroxide (H_2_O_2_) [49,50]. Furthermore, this residual chemical suppression may be synergistically amplified by intense nutrient competition as the starved microbes scavenge for essential amino acids [51,52].

### 4.5. CFS Assays Unmask Hierarchical Mechanisms of Bioprotection

To systematically separate these multi-mechanistic pathways, targeted chemical and enzymatic treatments on cell-free supernatants (CFSs) were utilized. The resulting data strongly indicated definitively that bioprotection relies on a hierarchical, multi-hurdle defense system dictated by nutrient availability and ecological competition.

In the carbohydrate-rich matrix (BHI:MRS), CFS assays confirmed that environmental acidification serves as the foundational mechanism of *L. monocytogenes* suppression. For strains like DSM 20205 and the *Lpb. pentosus* isolates, the complete loss of inhibitory capacity following pH neutralization aligns precisely with the extensive CFS literature, confirming that the accumulation of organic acids operates as the solitary active baseline defense for standard fermentative strains [53,54].

However, targeted catalase treatments suggested a profound mechanistic divergence, pointing to a potentially inducible oxidative defense system in the high-efficacy *Lc. lactis* isolates and elite *Lpb. plantarum* strains. While H_2_O_2_ is a known LAB metabolite, its direct contribution to pathogen suppression in standard CFS assays is frequently reported as highly variable [55,56,57].

These observations may account for this variability, suggesting that the accumulation of inhibitory H_2_O_2_ in these specific strains may be inducible. The synergistic oxidative burst, evidenced by the significant increase in pathogen recovery when catalase was added to neutralized co-culture CFSs, was not observed in the corresponding monocultures. This observation suggests that the catalase-sensitive metabolite is not passively accumulated as a background byproduct, but rather actively triggered by the severe ecological stress of direct competition with *Listeria*, a pattern analogous to the competition-induced metabolic responses described by Peng et al. [58]. However, direct chemical quantification of H_2_O_2_ is necessary to confirm this competition-induced accumulation.

Finally, under nutrient-depleted conditions where acidogenesis failed, the universal restoration of pathogen growth following proteinase-K digestion suggested the constitutive secretion of protease-sensitive compounds, pointing to bacteriocin-like inhibitory substances (BLIS). Enzymatic neutralization via protease treatment is an established method for identifying proteinaceous antimicrobials [59,60]. Unlike the inducible catalase-sensitive activity, this protease-sensitive inhibition was identical across both monocultures and co-cultures. This suggests a constitutive secretion of proteinaceous inhibitors, independent of the presence of the pathogen. By maintaining this proteinaceous activity, the LAB strains appear to possess an intrinsic mechanism to limit pathogen proliferation even when primary glycolytic pathways are restricted by nutritional deficits.

Several limitations should be considered when interpreting these results. All experiments were conducted in liquid broth at 30 °C, a condition selected to support near-optimal growth of both partners and to resolve strain-level differences in competitive fitness; bioprotective performance in structured food matrices and at refrigeration temperatures, where LAB acidification is disproportionately slowed, cannot be extrapolated directly and requires dedicated validation. Furthermore, a single *L. monocytogenes* strain (Scott A, serotype 4b) was used as the target organism. Because susceptibility to LAB-derived antimicrobials—particularly bacteriocins and organic acids—can vary significantly among different *L. monocytogenes* strains and serotypes, the specific inhibitory dynamics and clearance thresholds observed here cannot be broadly generalized to the entire species. While Scott A is a well-characterized clinical isolate widely used as a benchmark in predictive microbiology, future challenge studies must employ multi-strain cocktails spanning prevalent food-associated serotypes (e.g., 1/2a, 1/2b, and 4b) to properly account for intra-species variability and confirm broad-spectrum bioprotective efficacy. Moreover, while the multivariate clustering analysis provided a useful descriptive method to compare kinetic profiles, the small sample size (*n* = 9) limits the broader biological significance of these specific groupings. Evaluating a larger, more diverse library of LAB isolates is required to validate these functional clusters on a population level. Additionally, the contribution of hydrogen peroxide was inferred from catalase sensitivity rather than direct quantification, and proteinaceous activity from proteinase-K sensitivity; neither the peroxide concentrations nor the identity of the inhibitory peptides was determined, and the latter are therefore described as bacteriocin-like inhibitory substances.

## 5. Conclusions

Bioprotective efficacy against *L. monocytogenes* in this strain panel appeared to be primarily driven by the speed of acidification rather than by LAB growth rate or inoculum size alone. Strains that lowered the pH within the first 6 h of co-culture, the three *Lc. lactis* isolates and *Lpb. plantarum* DSM 20205, restricted pathogen growth by 31–41%, consistent with an early onset of the “Jameson effect”. Under carbohydrate limitation, this primary mechanism largely failed for the *Lactiplantibacillus* isolates, while *Lc. lactis* BL1 and BL8 retained partial activity, suggesting the presence of acid-independent mechanisms. Cell-free supernatant assays pointed to a potentially layered system: organic acids acting as the baseline under nutrient-rich conditions, a catalase-sensitive activity appearing specifically under competition, and a protease-sensitive activity present across all isolates irrespective of carbohydrate availability. For the selection of bioprotective cultures, these results argue for screening candidates on acidification kinetics together with acid-independent activity, rather than on end-point inhibition alone. However, while this study provides fundamental mechanistic insights, the practical implications for commercial biopreservation must be interpreted cautiously. Because these dynamic interactions were evaluated under near-optimal in vitro conditions, validation in structured food matrices at chill temperatures, where LAB acidification is inherently slowed, alongside the direct quantification of hydrogen peroxide and characterization of the protease-sensitive compounds, remain essential next steps.

## Figures and Tables

**Table 1:** Lactic acid bacteria strains used in this study.

LAB Strains	Source of Isolation
*Lpb. plantarum* ssp. *plantarum* DSM 20205	DSMZ (ATCC 8014)
*Lpb. plantarum* 2640	Feta cheese (ACA-DC)
*Lpb. plantarum* BS11	Fresh feta cheese
*Lpb. plantarum* BS14	Fresh feta cheese
*Lpb. pentosus* 281	Fermented black olives
*Lpb. pentosus* AS15	Fresh feta cheese
*Lc. lactis* subsp. *lactis* BL2	Mature feta cheese
*Lc. lactis* subsp. *lactis* BL8	Mature feta cheese
*Lc. lactis* subsp. *lactis* BL1	Mature feta cheese

**Table 2:** Zones of inhibition (mm ± SD) of LAB strains against *Listeria monocytogenes* Scott A. Values are represented as mean ± standard deviation. Different superscript letters (^a–d^) within the same column indicate statistically significant differences (*p* < 0.05).

LAB Strains	Inhibition Zone (mm)
*Lpb. plantarum* ssp. *plantarum* DSM 20205	25.0 ± 1.4 ^b^
*Lpb. plantarum* 2640	21.0 ± 0.8 ^a^
*Lpb. plantarum* BS11	21.0 ± 0.00 ^a^
*Lpb. plantarum* BS14	21.5 ± 2.1 ^ab^
*Lpb. pentosus* 281	17.3 ± 0.50 ^d^
*Lpb. pentosus* AS15	16.7 ± 0.58 ^d^
*Lc. lactis* subsp. *lactis* BL2	13.7 ± 0.58 ^c^
*Lc. lactis* subsp. *lactis* BL8	14.0 ± 1.00 ^c^
*Lc. lactis* subsp. *lactis* BL1	13.5 ± 0.71 ^c^

## Data Availability

The original contributions presented in this study are included in the article/Supplementary Materials. Further inquiries can be directed to the corresponding author.

## Supplementary Materials

The following supporting information can be downloaded at https://www.mdpi.com/article/10.3390/microorganisms14102331/s1. Table S1: Maximum growth rate of LAB during co-culture, onset of pH decline (pH_t_lag) of LAB in co-culture, strain inhibition and max growth rate of *L. monocytogenes* during co-culture. Table S2: Maximum growth rate of LAB during co-culture, strain inhibition and max growth rate of *L. monocytogenes* during co-culture. Figure S1: Mechanistic profiling of LAB inhibitory activity in nutrient-rich media (BHI:MRS 1:1). Bars indicate the final change in *L. monocytogenes* optical density (ΔOD_600_) after 24h exposure to differentially treated CFSs from (A) co-cultures and (B) monocultures. Different lowercase letters above bars represent statistically significant differences (*p* < 0.05) within each strain’s treatment group. Figure S2: Mechanistic profiling of LAB inhibitory activity in nutrient-depleted media (TSB w/o glucose). Bars indicate the final net change in *L. monocytogenes* optical density (ΔOD_600_) after 24 h of exposure to differentially treated cell-free supernatants (CFSs) harvested from (A) co-cultures and (B) monocultures. Different lowercase letters above the bars represent statistically significant differences (*p* < 0.05) within each strain’s specific treatment group.
