# Supplementary material for: Strain-Specific Antimicrobial Mechanisms of Lactic Acid Bacteria Against Listeria monocytogenes Scott A Under Nutrient-Rich and Carbohydrate-Limited Conditions
Source: Microorganisms. 2026 Oct 13;14(10):2331. doi: 10.3390/microorganisms14102331 (PMC13707551; doi:10.3390/microorganisms14102331)
Supplement: Supplementary file 1 [file microorganisms-14-02331-s001.zip › Supplementary Figure S1.pdf]

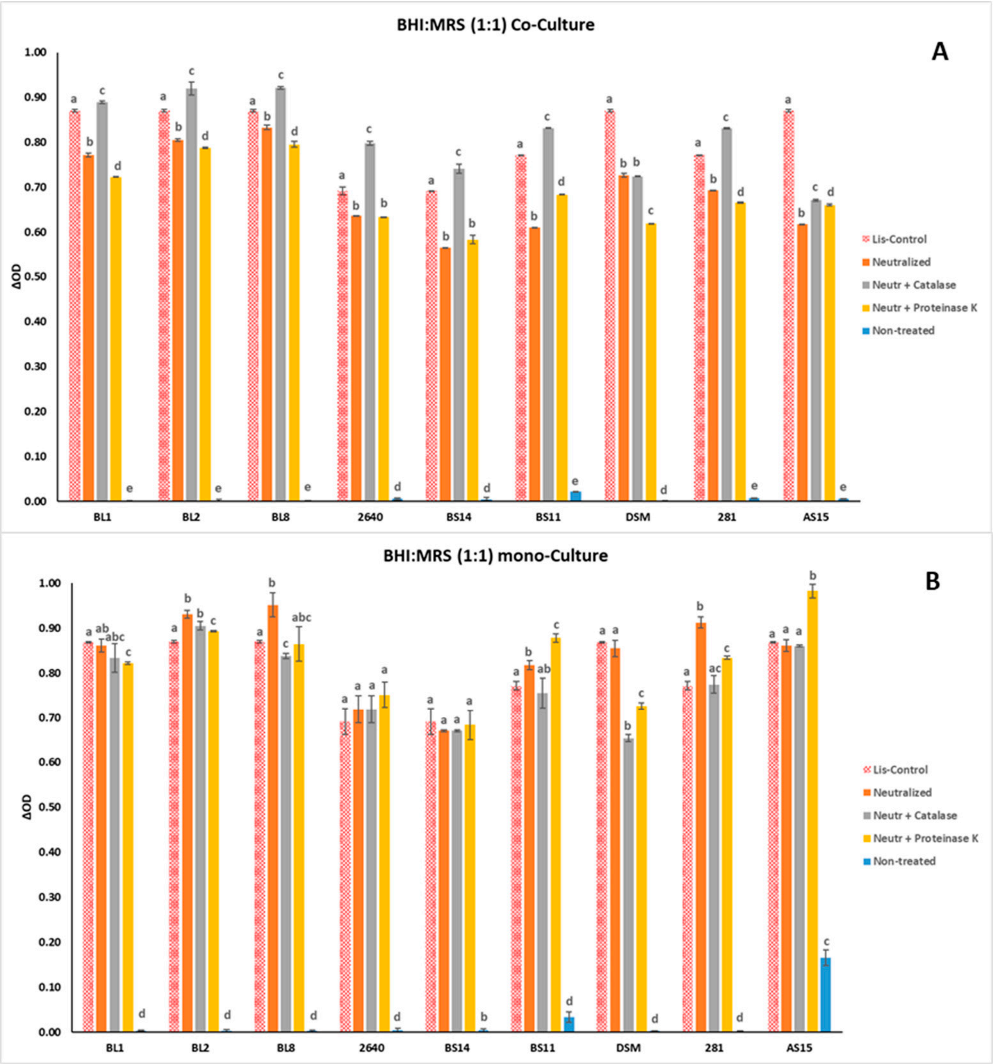

Figure S1. Mechanistic profiling of LAB inhibitory activity in nutrient-rich media (BHI:MRS 1:1). Bars indicate the final change in *L. monocytogenes* optical density (ΔOD_{600}) after 24h exposure to differentially treated CFSs from (A) co-cultures and (B) mono-cultures. Different lowercase letters above bars represent statistically significant differences ($p<0.05$) within each strain's treatment group.
