# Supplementary material for: Strain-Specific Antimicrobial Mechanisms of Lactic Acid Bacteria Against Listeria monocytogenes Scott A Under Nutrient-Rich and Carbohydrate-Limited Conditions
Source: Microorganisms. 2026 Oct 13;14(10):2331. doi: 10.3390/microorganisms14102331 (PMC13707551; doi:10.3390/microorganisms14102331)
Supplement: Supplementary file 1 [file microorganisms-14-02331-s001.zip › Supplementary Figure S2.pdf]

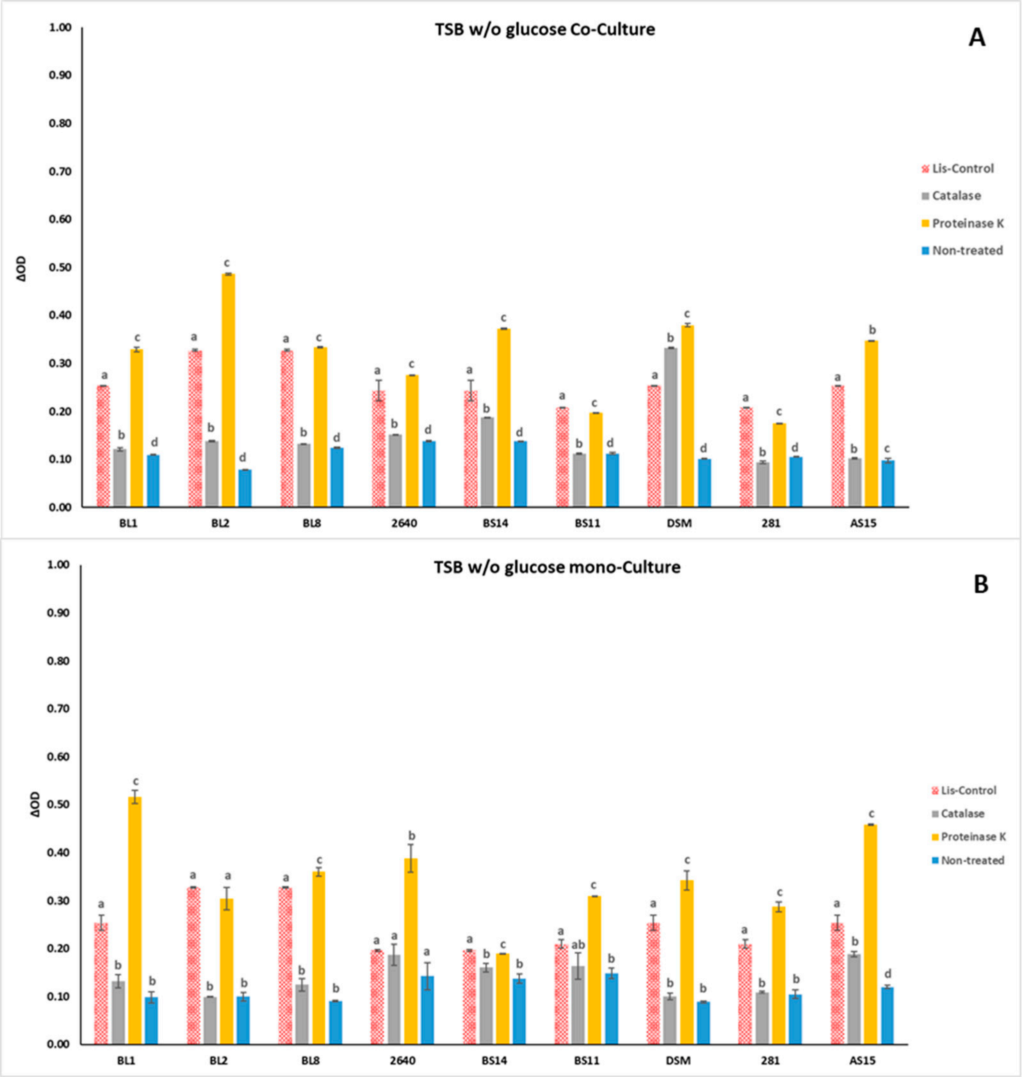


**Figure S2**. Mechanistic profiling of LAB inhibitory activity in nutrient-depleted media (TSB w/o glucose). Bars indicate the final net change in *L. monocytogenes* optical density ($\Delta OD_{600}$) after 24 h of exposure to differentially treated cell-free supernatants (CFSs) harvested from (A) co-cultures and (B) mono-cultures. Different lowercase letters above the bars represent statistically significant differences ($p < 0.05$) within each strain's specific treatment group.
