# Supplementary material for: Strain-Specific Antimicrobial Mechanisms of Lactic Acid Bacteria Against Listeria monocytogenes Scott A Under Nutrient-Rich and Carbohydrate-Limited Conditions
Source: Microorganisms. 2026 Oct 13;14(10):2331. doi: 10.3390/microorganisms14102331 (PMC13707551; doi:10.3390/microorganisms14102331)
Supplement: Supplementary file 1 [file microorganisms-14-02331-s001.zip › Supplementary Table S1.pdf]

Table S1. Maximum growth rate of LAB during co-culture, onset of pH decline (pH_t_lag) of LAB in co-culture, strain inhibition and max growth rate of L. monocytogenes during co-culture.

LAB Strains	Max Growth Rate of LAB during Co-Culture (h-1)	pH_t_lag (h)	Strain Inhibition (%)	Max Growth Rate of L. monocytogenes during Co-Culture (h-1)
*Lpb. plantarum*DSM20205	0.84	6	35.94	0.305
*Lpb. plantarum*ACA-DC 2640	0.91	6	31.12	0.306
*Lpb. plantarum*BS11	0.748	8	19.89	0.333
*Lpb. plantarum*BS14	0.84	10	13.16	0.338
Lpb. pentosus B281	0.656	10	16.58	0.326
Lpb. pentosus AS15	0.737	8	16.26	0.339
Lc. lactis subsp. *lactis* BL2	1.14	6	35.19	0.234
Lc. lactis subsp. *lactis* BL8	1.14	6	40.53	0.203
Lc. lactis subsp. *lactis* BL1	1.25	6	37.33	0.191
