# Supplementary material for: Strain-Specific Antimicrobial Mechanisms of Lactic Acid Bacteria Against Listeria monocytogenes Scott A Under Nutrient-Rich and Carbohydrate-Limited Conditions
Source: Microorganisms. 2026 Oct 13;14(10):2331. doi: 10.3390/microorganisms14102331 (PMC13707551; doi:10.3390/microorganisms14102331)
Supplement: Supplementary file 1 [file microorganisms-14-02331-s001.zip › Supplementary Table S2.pdf]

Table S2. Maximum Growth Rate of LAB during Co-Culture, Strain Inhibition and Max Growth Rate of L. monocytogenes during Co-Culture

**LAB Strains**	**Max Growth Rate of *Listeria* during Co-culture (h^{-1})**	**Strain Inhibition (%)**	**Max Growth Rate of LAB during Co-culture (h^{-1})**
*Lpb. plantarum*ssp. plantarum DSM20205	0.45	5.09	0.35
*Lpb. plantarum*ACA-DC 2640	0.505	4.33	0.245
*Lpb. plantarum*BS11	0.43	1.27	0.365
*Lpb. plantarum*BS14	0.41	2.76	0.44
Lpb. pentosus B281	0.745	4.24	0.295
Lpb. pentosus AS15	0.45	1.09	0.305
Lc. lactis subsp. *lactis* BL2	0.39	4.26	0.525
Lc. lactis subsp. *lactis* BL8	0.805	16.23	0.535
Lc. lactis subsp. *lactis* BL1	0.35	16.88	0.535
